# Clinical strategies for reducing firearm suicide

**DOI:** 10.1186/s40621-021-00352-8

**Published:** 2021-10-04

**Authors:** Rocco Pallin, Amy Barnhorst

**Affiliations:** 1 University of California Firearm Violence Research Center at UC Davis, 2315 Stockton Blvd, Sacramento, CA USA; 2 Department of Emergency Medicine, UC Davis School of Medicine, 2315 Stockton Blvd, Sacramento, CA USA

**Keywords:** Firearm violence, Clinical interventions, Firearm storage, Firearm suicide, Prevention, Lethal means safety, Lethal means counseling

## Abstract

Suicide is complex, with psychiatric, cultural, and socioeconomic roots. Though mental illnesses like depression contribute to risk for suicide, access to lethal means such as firearms is considered a key risk factor for suicide, and half of suicides in the USA are by firearm. When a person at risk of suicide has access to firearms, clinicians have a range of options for intervention. Depending on the patient, the situation, and the access to firearms, counseling on storage practices, temporary transfer of firearms, or further intervention may be appropriate. In the USA, ownership of and access to firearms are common and discussing added risk of access to firearms for those at risk of suicide is not universally practiced. Given the burden of suicide (particularly by firearm) in the USA, the prevalence of firearm access, and the lethality of suicide attempts with firearms, we present the existing evidence on the burden of firearm suicide and what clinicians can do to reduce their patients’ risk. Specifically, we review firearm ownership in the USA, firearm injury epidemiology, risk factors for firearm-related harm, and available interventions to reduce patients’ risk of firearm injury and death.

## Introduction

Suicide is a complex problem with psychiatric, socioeconomic and cultural roots and thus cannot be eliminated with one single solution. In light of high rates of firearm suicide in the USA, the high prevalence of household firearm ownership, and the importance of reducing access to the lethal means for suicide prevention, we review what is known about firearm ownership, firearm suicide epidemiology, and a range of clinical interventions available to better equip clinicians to reduce their patients’ risk of firearm suicide [1, 2]. We then provide a clinical example to illustrate the application of key points on clinician counseling to reduce access to firearms for suicidal persons.

## Background

Firearms are used in 5 % of suicide attempts but account for half of suicide deaths because they are the most lethal means: 90 % of suicide attempts with firearms result in death (compared to 53 % for hanging USA) [3]. Further, the majority of suicide attempts are impulsive, with little time passing between the decision to attempt and the attempt itself [4]. The odds of dying by suicide are more than 3 times higher for those with access to firearms compared to those without access [5]. Assessing a suicidal patient’s firearm access and working with the patient, loved ones, and other trusted partners to reduce access during a period of heightened risk may help reduce the chance of fatality [6, 7]. When collaboration with the patient is not possible, emergency or involuntary interventions may be necessary. A basic understanding of who owns firearms and who dies by firearm suicide in the USA may help clinicians take informed and respectful steps towards lethal means reduction for suicidal patients.

### Firearm ownership in the USA

Firearm ownership is common in the USA: one in three households has a gun. Gun owners come from diverse backgrounds and geographic areas and have varied reasons for owning firearms [8]. There are between 300 and 400 million civilian-owned firearms in the USA [8, 9]. Although that roughly equates to an average of one firearm for every US adult, the distribution of firearms varies substantially, both demographically and geographically. An estimated 22 % of American adults own firearms, and another 13 % live in homes with firearms but are not owners themselves [8]. Firearm ownership has become more concentrated in recent decades: in 1994, 25 % of Americans were firearm owners and collectively owned 192 million firearms, but now fewer Americans own firearms and each owner owns more firearms [8]. Just over half of the current civilian stock of firearms are long guns (i.e., shotguns or rifles) and 42 % are handguns (i.e., pistols, revolvers, or other handguns) [8].

In the USA, older, white males have the highest rates of firearm ownership. Owners disproportionately live in non-urban and southern regions of the USA [8]. Rates of firearm ownership among American veterans are higher than for the general public for both males and females. Nearly half of male veterans (47 %) and one-quarter of female veterans own firearms, compared with 32 % of males and 12 % of females in the general population [10].

There is variation in the characteristics of firearm owners and ownership-related practices, including types of firearms owned, reasons for ownership, means of acquisition, and storage practices. About half (48 %) of firearm owners own 1 or 2 firearms, but 8 % of owners own 10 or more firearms [8]. In the USA, self-protection is most often cited as a major reason for firearm ownership: about two-thirds of owners say they own a firearm to protect themselves. Fewer report hunting (approximately 40 %), sport shooting (approximately 30 %), and other reasons (e.g., collections, inheritance, owning for work) [8, 11]. These differences can and should inform suicide and injury prevention campaigns, safe firearm storage interventions, and targeted public health policy [12].

Firearm storage practices also vary widely, in part due to type(s) of firearm(s) owned and reasons for ownership. Nearly one-third of firearm owners report storing at least one gun loaded and unlocked—the least secure method of storage—and one-quarter report the most secure storage practice, i.e., keeping all guns locked up and unloaded [13]. Despite widespread promotion of keeping guns locked up and unloaded in homes with children, an estimated 4.6 million children in the USA live in a home with at least one loaded and unlocked firearm [14]. Having a firearm in the home, regardless of storage method, increases household members risk of suicide and homicide [5]. Evidence suggests that keeping firearms loaded and not locked up is associated with increased risk of suicide, unintentional injury, and use of firearms by children in the home [15–18]. Further studies on the association between firearm storage practices and injury outcomes among all populations are needed.

Keeping firearms unloaded and locked up so that they are inaccessible to those who should not have access has been endorsed broadly for prevention of firearm-related harm. Many firearm owners (58 %) believe that having a firearm at home makes their home safer, despite evidence to the contrary [5, 19, 20]. Owners who believe a gun makes their home safer are more likely to store their firearms in the least secure manner (i.e., loaded and not locked up) [19].

An estimated 1 million Americans become new gun owners every year, and research suggests a substantially larger increase during the COVID-19 pandemic [21, 22]. Newer owners tend to be younger, live with younger children, identify as liberal, and more often report personal protection as their reason for acquiring a gun [21]. They may be less experienced in safe handling and storage practices. As both ownership and risk are dynamic, repeated assessment of risk for harm and of access to firearms when someone is at increased risk may be important for reducing injuries.

Firearms are readily available to Americans, whether or not they have them in their own home. The USA has 1.21 civilian-owned guns per capita, more than twice that of next-ranking Yemen (0.53 guns per capita). The USA number of guns per capita is six times the average rate of other OECD nations [9]. Regardless of whether they live in a home with them, many American adults have had exposure to firearms: 72 % have shot gun(s), two-thirds have lived in homes with gun(s) at some point, and nearly half grew up in households with gun(s) [11].

### US firearm suicide epidemiology

Firearm suicide is a major public health issue in the USA. In 2019, 39,707 people in the USA died from firearms, 60 % (23,941) of them from suicide [23]. Suicide by any method was among the top eight causes of death for Americans 10–64 years of age and the second-leading cause for those ages 10–34 [23]. In 2019, half of suicides nationwide involved firearms [23]. Though public mass shootings receive much media and public attention, deaths from mass shootings account for less than 1 % of all deaths from firearms [23, 24].

The US population firearm suicide rate has increased 24 % in the last two decades [23]. Teen suicide rates have increased more than those for any other age group in the last decade, with especially increasing rates among Black teens [23, 25, 26].

Some demographic groups in the USA experience disproportionately high rates of firearm suicide, especially middle-aged and older white men. The age-related increase in risk of firearm suicide for white males is particularly evident among those over age 70 [23]. In contrast to white males, rates of firearm suicide for Black and Hispanic males peak around age 20 and then largely decrease with age. Rates of firearm suicide for white males exceed those for black and Hispanic males throughout the lifespan. The same is true for females: rates for whites exceed those for Black and Hispanic females throughout the lifespan. For the age group in which white females have the highest firearm suicide rate (ages 50 and 54), the male rate is nearly five times as high.

The USA has uniquely high rates of firearm suicide: eight times the aggregate rate of other high-income countries [27]. This is not because Americans are more suicidal; the US total suicide rate (suicides by all methods) is comparable to rates in other high-income countries. Just 5 % of suicides in the countries of comparison were by firearm, and half of US suicides were by firearm. Research suggests that prevalence of firearms affects suicide rates and that in areas where more firearms are available, firearm suicide and total suicide rates are increased [28–30].

**Fig. 1:**
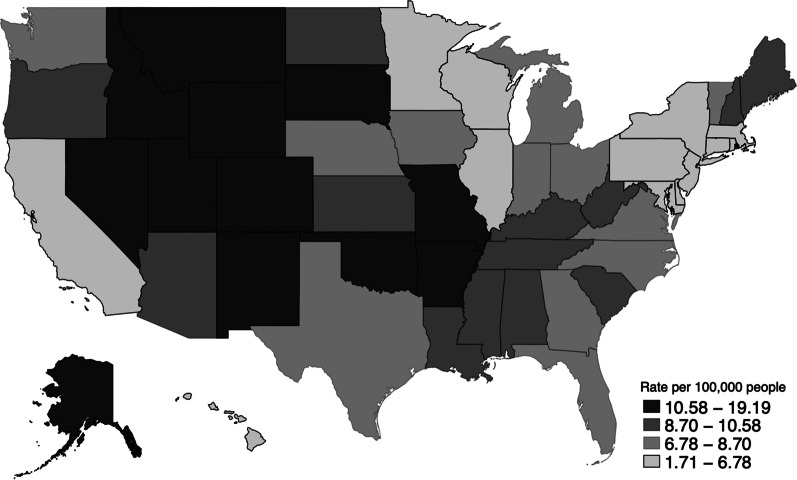
Age-Adjusted Firearm Suicide Rates by US State, 2019. Data from CDC WISQARS [23].

US firearm suicide rates vary dramatically across states, ranging from 1.7 to 19.2 per 100,000 residents in New Jersey and Wyoming, respectively (Fig. 1). In rural areas, rates of firearm suicide rates far outpace those of firearm homicides. Suicide rates are especially high in the intermountain states, the Ozarks, Appalachia, and Alaska [23].

According to emergency department data, over 85,000 non-fatal firearm injuries occur in the USA each year [31]. These data suggest that half are unintentional injuries and the remainder are primarily assault-related (41 %). 3 % are from suicide attempt, as the vast majority of suicide attempts with firearms result in fatality [31]. There is some concern, however, about the possibility of mis-classifications of intent in this data (specifically, over-classification of unintentional injuries and under-classification of assault-related injuries) [32].

People who are not firearm owners nor victims of firearm violence themselves also experience firearm-related harm. 44 % of Americans know someone who has been shot [11]. Research has found a link between youth exposure to firearm injury (suicide, assault, and mass shootings) and high rates of future injury and post-traumatic stress symptoms [33]. Other long-term physical, financial, social, and psychological effects of firearm injury and death exist for victims, families and friends, and communities [34].

## In clinical practice

The clinical approach for helping to reduce access to firearms for patients who are suicidal can be risk based, individualized, respectful, and rooted in the principles of harm reduction. The appropriate intervention will depend on the risk factors present, the level of risk, the person’s ability to collaborate to reduce access, and the nature of the suicidal person’s access to firearms (e.g., personal ownership vs. access to someone else’s firearms).

### Risk factors for firearm suicide

Individual-level risk factors for firearm suicide include alcohol and substance misuse, dementia or other cognitive impairment, prior suicide attempt, chronic pain, and serious and poorly controlled mental illness [3, 4, 35–44]. As many as 10 % of American adults self-report both patterns of impulsive, angry behaviors and access to firearms [45]. Additionally, research has found a relationship between ownership and prevalence of risky drinking behaviors, such as binge drinking and of driving under the influence, and suggests that firearm owners who misuse alcohol may be at even greater risk of firearm violence or suicide [46].

Risk of suicide for heavy alcohol consumers is as much as five times that of social drinkers [47]. Forty percent of patients who seek treatment for alcohol dependence report at least one prior suicide attempt [48]. A 2013 national study found that one-third of those who died by suicide tested positive for alcohol and that those who used firearms were more likely to be intoxicated at time of the act compared to those who used other, less lethal methods [39].

Firearm access poses risk for people with dementia: in the USA, 60 % of persons with dementia live in a home with a firearm, and firearms are the most common means of suicide among persons with dementia [49, 50]. Research has found that risk of suicide may be especially elevated in the period after an older adult receives a dementia or mild cognitive impairment diagnosis [43]. Talking with caregivers of patients with dementia about the risk of firearms remains rare [51], and experts suggest that conversations about firearms and dementia should parallel those about driving and dementia [52].

Mental illness also plays a substantial role in self-harm and suicide. Research suggests that depression, bipolar disorder, and other psychiatric conditions are associated with 45–90 % of suicides by any means in the USA [53–55]. The range of estimates is wide because research variably uses data from pre-existing records or from psychological autopsies (which pose methodological challenges).

Veterans may be especially at risk of firearm suicide, as they have high rates of firearm ownership, may not store all firearms securely, and disproportionately experience suicidal thoughts compared to non-veterans [56, 57]. Factors besides mental illness contribute to their risk. Among a large cohort of veteran suicide decedents, 45 % had no mental health or substance use diagnosis, and those without such diagnosis were significantly more likely to die by firearm suicide than by other means [58]. Efforts to raise awareness of risk, discuss the importance of suicide risk assessment regardless of the presence of a diagnosis, create guidance for acceptable counseling on reducing access to firearms for those at risk of self-harm, and increase access to mental health services are underway at the VA [59], and additional work is needed to address firearm suicide specifically among veterans [60].

Regardless of demographic or individual risk factors, patients who make threats of suicide or who have suicidal ideation, including patients whose suicidal ideation may not be acute, but instead is intermittent, situational, or chronic, are at increased risk of death if they have access to a firearm [5, 30, 61]. Limited evidence suggests that adolescents who die by firearm suicide are significantly more likely to live in homes with firearms and that firearms used for adolescent suicides are most often owned by parents [62, 63]. As access to a firearm increases the risk of suicide by more than three times, clinician evaluation of any suicidal patient should involve a careful assessment of their access to firearms [5].

### Risk identification and clinical intervention

After a clinician has evaluated risk and determined that firearm access is clinically relevant, it is recommended they plan a tailored conversation about why firearm access is important and what can be done if the patient does have access [2, 64]. When asking questions about firearms in the home, it is important to use neutral, non-judgmental language and relate the conversation clearly to the health and safety of the patient and others in the home [65]. Using phrasing that normalizes talking about access to firearms and puts the conversation in the context of risk may be most effective. Experts suggest that assuming a patient at risk has a gun (rather than leading the conversation by asking about gun ownership directly) may help a clinician to make clear that they respect the patient’s decision to own firearms [66], though evidence on best practices is lacking.

The clinician’s overall goal should be to reduce access to firearms for the person at increased risk, usually by counseling on firearm storage practices or temporary transfers of firearms that render firearms inaccessible to those at risk. The principles of harm reduction apply here; the goal is not necessarily to stop the behavior but to mitigate the associated risks. Any steps in that direction are progress [67]. Knowing the types of firearms in the home and reasons for ownership may help clinicians make appropriate recommendations, but this knowledge is not necessary in order to counsel on firearm storage and reduce risk. Clinicians can present storage options that are amenable to owners of different types of firearms and those who own for different reasons without engaging in detailed discussions on reasons for ownership.

### Considerations for conversations about firearm access

A basic understanding of the different types of guns, the reasons for ownership and use, and various methods of storage will help clinicians have effective and collaborative conversations about reducing access to firearms to reduce suicide risk. Knowing who in the household owns the gun(s), to best understand who has and makes decisions about firearm access and storage, may be helpful for recommending the most appropriate interventions.

When engaging in clinical counseling for firearm injury prevention, clinicians can consider that many Americans value firearm ownership and such ownership may be part of a patient’s identity [65, 68, 69]. Messaging should therefore acknowledge and respect the role firearms can play in people’s identities and acknowledge that many firearm owners take firearm injury prevention, safety, and responsible firearm storage seriously [65, 66]. Conversations about firearm access may be perceived as political, but politics has no place in these conversations [70]. If politics comes up, the clinician should redirect the conversation to reinforce the shared goal of reducing access for those at risk. Research suggests that clinicians are not the most acceptable messengers of firearm storage information [71, 72].

Nonetheless, patients are generally receptive to provider-initiated conversations about firearms and clinicians are trained for and accustomed to having conversations with patients about safety and risks in the home. A majority of American adults find such conversations appropriate “in general,” including 54 % of firearm owners [73]. Acceptability of these conversations may increase when a patient has access to a firearm and someone in the home is at increased risk of firearm injury, such as a person with suicidal ideation or a child or teen [74]. Just 8 % of American adults living in homes with firearms, however, have ever had a clinician initiate a conversation about firearms [75]. Research suggests that veterans, who more often own firearms and more often have risk factors for suicide, are also supportive of health care interventions to reduce access to firearms in times of acute risk for self-harm [76].

Medical and mental health care providers are allowed to discuss firearms with patients; no state or federal statutes prevent these discussions when access to firearms is relevant to the health of the patient or to someone else [1]. Although a 2011 Florida statute attempted to restrict provider conversations about firearms with patients unless the physician felt, in good faith, the conversation was relevant to someone’s health, it was struck down in court in 2017 for restricting health care providers’ First Amendment rights [1].

### Lethal means safety

Limiting access to lethal methods of attempt, including firearms, may be a promising intervention for preventing suicide. There is evidence that population-level interventions that reduce suicidal individuals’ access to the most lethal means of suicide reduces suicides [77]. Healthcare providers can play an important role in lethal means reduction. Suicidal crises can be difficult to predict and can become serious quickly. Research with survivors of suicide attempts suggests the decision to end one’s life is often impulsive: as many as 70 % of people who made near-lethal attempts made the decision to attempt in less than one hour, and 24 % made the decision to attempt in less than five minutes [78]. Though prior suicide attempt is a significant risk factor for future suicide, most survivors of suicide attempts do not go on to die by suicide [4]. If a clinician can help get someone through the acute suicidal crisis and a lethal attempt can be prevented, a life may be saved.

For patients at risk, there is a range of lethal means safety for prevention of firearm suicide. Generally, this includes two counseling and two emergency clinical interventions: counseling on firearm storage, counseling on temporary firearm transfers, mental health holds, and extreme risk protection orders (ERPOs).

Evidence suggests that counseling on firearm storage can influence firearm storage practices [79]. Secure storage that removes access to firearms for those at risk is critical for suicide prevention and in some cases may be accomplished by storing firearms unloaded and locked up using a locking device, such as a cable lock or gun safe.

Clinicians should consider, however, that reasons for ownership may affect willingness to store guns locked up and unloaded. For example, if a patient living with an at-risk person owns a gun for self-protection and feels the need to have immediate access, the recommendation that all firearms are stored unloaded, locked up, and separate from ammunition may be unacceptable. Taking a harm reduction approach, the clinician could recommend a solution that would allow quick access to the firearm but removes access to the person at risk, such as a biometric lockbox (e.g., one that uses fingerprint technology) that opens instantaneously and only for authorized persons. A collaborative approach that explores the options a patient finds acceptable may be most effective for reducing access for those at risk.

If a gun owner themselves is at increased risk, rather than someone in the home who does not personally own firearms (and therefore control how firearms are stored), reducing access to lethal means without removing firearms from the home is more difficult. In such cases, rather than promoting use of locking devices and keeping firearms unloaded, appropriate options may include changing codes or keys to storage devices and giving access only to trusted family or friends and removing the firing pin(s) or otherwise disassembling the firearm(s) to render them temporarily unusable, increasing the time it would take to use them in a suicide attempt [80].

Storing firearm(s) out of the home is the preferred method for reducing access to firearms for the duration of a crisis [77]. Options for voluntary, temporary firearm transfer vary locally and by state and may include temporarily transferring firearms to a trusted individual like a friend or family member living outside the at-risk person’s household; storing firearms at a gun shop or range or with local law enforcement; or selling the firearm(s). Firearm owners or caregivers may prefer solutions that involve family members or gun shops/ranges over options for temporary storage than those involving law enforcement agencies [81]. When discussing temporary transfer, using the words “temporary” and “voluntary” may help. “Temporary” emphasizes that such transfers are not permanent and also serves as a reminder of the often temporary nature of suicidal crises; “voluntary” may help the patient retain a sense of agency [65]. As with keeping firearms stored locked up and unloaded, consider that willingness to temporarily transfer firearms may be affected by the perception that doing so would make the home more vulnerable to outside threats [82]. Clinicians should be prepared to discuss balancing risk-reducing storage strategies with perceived decreases in feelings of personal safety for patients who own firearms for self-protection.

Resources for patients and patients’ families detailing options for storage outside the home exist but are inconsistently available. In a growing number of states, including Colorado, Maryland and Washington, researchers have developed Gun Storage Maps, where community members can find local options for voluntary, temporary firearm storage with retailers, ranges, and law enforcement agencies [83, 84]. Creators of the Maryland map have published details on the process and barriers to creating such a tool [85].

In some states, legal considerations for temporary transfers may warrant exploration. For example, in some places, temporary transfer of firearms is not permitted unless a background check is performed and the temporary recipient of the firearm is deemed legally able to possess firearms. These practices are in flux, however. For example, in California, strict temporary transfer policies were relaxed in January 2020 to allow immediate family members to temporarily receive and hold firearms without a background check if for the express purpose of prevention of self-harm, with some restrictions [86]. Policy on criteria for returning firearms is lacking, especially issues of who decides when someone who has gone through a crisis should have firearms returned to them and who is liable should a bad outcome take place [87].

### Emergency intervention

In some situations, an involuntary mental health hold may be an appropriate intervention for patients at acute risk of suicide or violence. Most states allow a person who poses a danger to themselves or someone else to be detained involuntarily for the purpose of psychiatric evaluation. Depending on the state, such a hold can be initiated by law enforcement, mental health professionals, physicians or family members [88]. While these holds may be an effective way to remove a person from an acutely threatening or dangerous situation, they do not guarantee that the person will receive treatment that eventually reduces their risk, nor that they will result in a prohibition on purchasing or owning a firearm upon hold expiration or in the future. Federal law prohibits people who have been “committed to a mental institution” from firearm ownership, but that criterion is not met until the civil commitment hearing, which can happen up to a few weeks into a psychiatric hospitalization [89].

For people who are at risk of suicide or other violence but do not meet involuntary commitment criteria and are not willing to temporarily relinquish their firearms, a growing number of states are passing and implementing risk-based firearm removal laws such as extreme risk protection orders (ERPOs), commonly referred to as “red flag laws.” Generally, ERPOs provide an individualized tool for temporary removal of firearm(s) and prohibition on purchase for individuals who pose a risk to self or others but who have not committed an offense prohibiting them from possession or purchase and for whom other interventions for risk reduction have been exhausted or are not appropriate. In Connecticut, such a law has been shown to be effective for suicide prevention, with an estimated one suicide prevented for every 10–20 firearm removal actions [90, 91]. In many states, law enforcement, family, and household members can petition for ERPOs. As of writing, 19 states and the District of Columbia have such emergency risk reduction policies.

In a few states (MD, CT, HI), clinicians can directly petition for ERPOs but elsewhere, a clinician may provide evidence of dangerousness to a petitioner. Experts have noted concerns about clinicians directly petitioning for ERPOs [92]. Nonetheless, ERPOs focus on risk and reducing access to lethal means, key principles of healthcare and public health [93] and clinician awareness of this tool may be useful. For example, clinicians are uniquely situated to identify patients at risk of self-harm. If a clinician is seeing a patient at acute risk for harm to self or others, the Health Insurance Portability and Accountability Act (HIPAA) permits disclosure of patient information when such disclosure is “necessary to prevent or lessen a serious and imminent threat to the health or safety of a person or the public” and “is to a person or persons reasonably able to prevent or lessen the threat” [94].

## A clinical example

With the following clinical example, we explore ways to help reduce an at-risk patient’s chance of attempting firearm suicide.

A 67-year-old man comes to his annual physical with his son. His son has become increasingly concerned about his father’s state of mind since his mother (the man’s wife) died 10 months ago. He has noticed his father’s drinking increases and that when he drinks, he seems to get more depressed.

The physician sees the father on his own and he admits that he has felt increasingly alone and isolated this past year. He is not a daily drinker, but he admits that, once a week or so, he has a few beers, often drinking more than he intends to. When he does drink, his depression gets worse, and sometimes he wonders if the world would be a better place without him. When he is not drinking, he denies ever feeling suicidal, but admits he has been pretty down, to the point that he doesn’t go out and see friends, or have the energy to do much. His children have recommended he seek counseling but he is reluctant. The physician talks with him about the possibility engaging in therapy and potential benefits. When the son comes back in at the end of the visit, he asks, “Did you tell her about your guns, dad?”

Access to firearms is clinically relevant in this case. Though the patient’s risk of suicide may only be moderate in the clinical encounter, the physician may be concerned that risk increases significantly when he is home drinking alone. Ideally, the patient would be willing to temporarily transfer his guns to a family member or friend for safekeeping—that person would hold on to the guns until his drinking was diminished and his grief and depression had subsided. If he were not willing to temporarily part with his guns, even separating the guns from the ammunition and locking them both, to create impediments to an impulsive firearm suicide attempt, may be a first step.

In this encounters, the patient does not appear to meet criteria for an involuntary intervention. If, however, he appeared to be at high enough risk of suicide that he required inpatient mental health treatment and was unwilling to participate in such treatment voluntarily, a mental health hold may be an appropriate clinical option. Once a mental health hold was in place, if the patient were released from the hospital before the civil commitment hearing, he would likely return home with his depression better treated, but the other circumstances unchanged, and his guns where he left them.

If the level of risk of firearm suicide was extremely elevated and the patient was unwilling to collaborate to reduce access to firearms (i.e., attempts to work with the patient to temporarily transfer the guns or safely store the guns in a way that reduces his access have failed, and no other options are available or appropriate), an ERPO may be warranted. This would likely involve talking about the possibility of an ERPO with the patient’s son or local law enforcement, either of whom could choose to act as petitioner for the order should they decide to proceed.

## Clinical takeaways

Firearm suicides can be reduced with risk evaluation and discussions about firearm access between clinicians and patients at risk. Considering the lethality of firearms in suicide attempts and establishing why firearms are clinically relevant once it is determined a patient is at risk of suicide are important when planning conversations about appropriate and acceptable methods to reduce access to lethal means and increase safety. Using a harm reduction approach, clinicians can have conversations about firearm access that are respectful, informed, and tailored to each patient’s needs. Further intervention may be appropriate if a patient is at imminent risk and counseling on firearm storage or discussions about temporary firearm transfer do not result in sufficiently reduced firearm access for the at-risk person. Learning more about firearms themselves, methods to store them such that they are inaccessible to those at risk, local options for temporary transfers, and gun ownership may help clinicians be more successful in reducing firearm suicide.

## Data Availability

Not applicable.

## Abbreviations

ERPO: Extreme risk protection order
HIPAA: Health Insurance Portability and Accountability Act
